# Pharmacological Activities of *Lonicerae japonicae flos* and Its Derivative—“Chrysoeriol” in Skin Diseases

**DOI:** 10.3390/molecules29091972

**Published:** 2024-04-25

**Authors:** Siu Kan Law, Xiao Xiao Wu, Zhou Jiang, Christy Wing Sum Tong, Wesley Yeuk Lung Chow, Dawn Ching Tung Au

**Affiliations:** 1 Department of Food and Health Sciences, The Technological and Higher Education Institute of Hong Kong, Tsing Yi, New Territories, Hong Kong, China; christytong@thei.edu.hk (C.W.S.T.); wesleychow@thei.edu.hk (W.Y.L.C.); 2 Laboratory Medicine Centre, Huazhong University of Science and Technology Union Shenzhen Hospital (Nanshan Hospital), Shenzhen 518056, China; wuxiaoxiaocuhk@gmail.com (X.X.W.); ethanzhou5188@163.com (Z.J.)

**Keywords:** *Lonicerae japonicae flos*, chrysoeriol, skin diseases, pharmacokinetic and pharmacodynamic activities, pharmacological function, cutaneous route

## Abstract

Chrysoeriol is an active ingredient derived from the Chinese medicinal herb (CMH) “*Lonicerae japonicae flos*” in the dried flower bud or bloomed flower of *Lonicera japonica* Thunberg. Dermatoses are the most common diseases in humans, including eczema, acne, psoriasis, moles, and fungal infections, which are temporary or permanent and may be painless or painful. Topical corticosteroids are widely used in Western medicine, but there are some side effects when it is continuously and regularly utilized in a large dosage. Chrysoeriol is a natural active ingredient, nontoxic, and without any adverse reactions in the treatment of dermatological conditions. Methods: Nine electronic databases were searched, including WanFang Data, PubMed, Science Direct, Scopus, Web of Science, Springer Link, SciFinder, and China National Knowledge Infrastructure (CNKI), without regard to language constraints. The pharmacological activities of chrysoeriol from *Lonicerae japonicae flos* to fight against skin diseases were explained and evaluated through the literature review of either in vitro or in vivo studies. Results: Chrysoeriol decreased the mRNA levels of proinflammatory cytokines IL-6, IL-1β, and TNF-α. These were transcriptionally regulated by NF-κB and STAT3 to combat skin inflammation. It also showed promising actions in treating many skin ailments including wound healing, depigmentation, photoprotection, and antiaging. Conclusion: The cutaneous route is the best delivery approach to chrysoeriol across the skin barrier. However, toxicity, dosage, and safety assessments of chrysoeriol in a formulation or nanochrysoeriol on the human epidermis for application in skin diseases must be further investigated.

## 1. Introduction

*Lonicerae japonicae flos* (called Jinyinhua in Chinese), is the flower or flower bud of *Lonicera hypoglauca* Miquel, *Lonicera confusa* De Candolle, or *Lonicera macrantha* (D.Don) Spreng, which belongs to the same family of Jinyinhua. This is recorded as the same herb in multiple versions of the Chinese Pharmacopoeia (ChP) [1].

*Lonicerae japonicae flos* is also termed “Rendong” in ancient books of traditional Chinese medicine (TCM). The Collective Notes to Canon of Materia Medica (around 480 to 498 AD) states the following: “It grows everywhere and is classified into liane, and does not fade over winter” [2]. This contains at least 212 biologically active ingredients, including 27 flavonoids, 83 iridoids, 17 triterpenoids, 41 organic acids (Table 1), and 45 other compounds [1,3], which have different pharmacological activities. Thus, it was widely used as a traditional Chinese medicine for several thousand years in China.

Dermatology is the medical specialty that deals with the study and treatment of different skin disorders, such as acne, psoriasis, eczema, moles, and fungal infections, which are temporary or permanent and may be painless or painful [4]. 

Acne is a disease of the pilosebaceous; it changes the keratinization pattern in the hair follicle leading to blockage of sebum secretion, which locks sebaceous glands and colonization [5]. Topical agents are the mainstay for the treatment of mild acne. Oral antibiotics for moderate acne and severe acne are treated with isotretinoin, which can lead to permanent remission [6].

Psoriasis is one of the most common dermatological conditions. It is a chronic inflammation of the skin, which is characterized by the formation of a rash with scaly, itchy patches over the body surface. Basically, this is related to the immune system wherein epidermal hyperplasia occurs with infiltration of immune cells [7]. Topical steroids, orally administered prednisolone, triamcinolone, and triamcinolone, were demonstrated to help reduce the epidermal keratinocytes in dermatoses for preventing psoriasis [8].

Eczema is an important example of chronic cutaneous inflammatory disease that affects more than 10% and 7.5% of adults in Western countries and China, respectively [9]. Generally, Western medicines use topical corticosteroids, glucocorticoids, antibacterial drugs, histamine_1_-receptor antagonists, immunomodulators, and other drugs to treat eczema [10]. However, the above Western medicines have side effects with drug resistance, leading to stunted growth, elevated blood sugar levels, and osteoporosis medical conditions [11]. Thus, these Western medicines are not the best choice for the treatment of skin diseases. In addition, they are required to be taken long term and administration cannot stop, otherwise, the skin disease can relapse. 

TCM is an alternative method of therapy for the prevention and treatment of dermatologic diseases. It is comparatively mild and has fewer adverse reactions, which is suitable for long-term usage [12]. TCM can treat the underlying causes of skin disease and state the reasons as well as classify them into different situations including diet, stress, allergies, genetics, toxins, etc. Treatment should be symptomatic. Probably, it is based on the TCM theory for “pattern identification and treatment”. Data, symptoms, and signs are collected in the four clinics of sight, smell, inquiry, and diagnosis [13]. 

Chinese medicinal herb (CMH) is commonly used as a combination to combat eczema because its pathogenesis is too complex for single-drug treatment. *Lonicerae japonicae flos* is a single herb that is always used in formulations to treat eczema. It contains at least 17.0% of all prescriptions from the herbal remedy [14]. Gu-Ben-Hua-Shi formula is an example. This formulation has seven herbs, including *Saposhnikoviae Radix, Coicis Semen*, *Curcumae Rhizoma*, *Atractylodis Macrocephalae Rhizoma*, *Rehmanniae Radix*, *Sophorae Flos*, *Atractylodis Rhizoma*, etc., except *Lonicerae japonicae flos* [15]. Therefore, *Lonicerae japonicae flos* is a type of meridian medicine, which can guide the active medicine in the prescription to reach the disease site or meridians, exerting the effectiveness of the medicine. Hence, the active ingredients from *Lonicerae japonicae flos* are worthy of attention and investigation. Chrysoseriol is one of the most important active ingredients in *Lonicerae japonicae flos*. Recently, Aboulaghras et al. considered the health benefits and pharmacological aspects of chrysoeriol, which has shown its promising potential to treat or prevent skin diseases, such as hypopigmentation disorder [16].

The present review article is mainly focused on skin diseases and aims to describe the sources, macroscopic features, and identification of *Lonicerae japonicae flos,* and its TCM theory. It also describes the extraction techniques of chrysoeriol from *Lonicerae japonicae flos* and explains its structure, which is related to pharmacological functions, pharmacokinetic and pharmacodynamic effects, and cutaneous delivery system in the treatment of skin diseases. 

## 2. *Lonicerae japonicae flos*

### 2.1. Sources

*Lonicerae japonicae flos* is primarily produced in Shandong, Shaanxi, Henan, and Hebei Provinces in China. Currently, Pingyi County, Linyi City, and Shandong Province are the largest production areas [17].

### 2.2. Macroscopic Features

**Figure 1:**
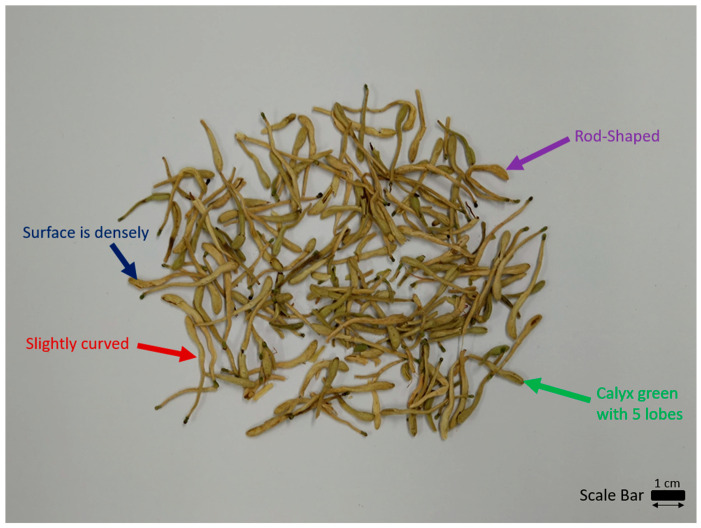
Macroscopic features of *Lonicerae japonicae flos*.

*Lonicerae japonicae flos* (Figure 1) is rod-shaped; thick at the top and thin at the bottom. It is slightly curved from 2 to 3 cm long. The upper diameter of *Lonicerae japonicae flos* is about 3 mm and it has a lower diameter of about 1.5 mm. It is a yellow–white or green–white color on a surface (the color gradually becomes darker after storage). This is densely covered with pubescence. Occasionally, its leaf-like bracts are seen. The calyx is green with five lobes at the apex, while the lobes are hairy and about 2 mm long. An open corolla is tube-shaped with a two-lipped apex which has five stamens and is attached to the tube wall. It is yellow with one pistil and the ovary is hairless [18].

### 2.3. Identification of Lonicerae japonicae flos 

This is a light yellowish brown or yellowish green color in powder. The outer surface of *Lonicerae japonicae flos* is covered with glandular hairs, which can be obconical, round, or slightly oblate, and made up of 4 to 33 cells with 2 to 4 layers in a diameter of 30 to 108 μm. Nonglandular trichomes are made up of one to five cells with a length of up to 700 μm. There are two types of nonglandular trichomes: (i) thick-walled nonglandular trichomes, single cells, up to 900 μm long, with fine wart-like or vesicular protrusions on the surface, some with threads; (ii) thin-walled nonglandular trichomes, single-celled, long, curved or wrinkled, with fine wart-like protrusions on the surface. The diameter of calcium oxalate clusters is 6 to 45 μm. Pollen grains are round or triangular with fine short spines and fine granular carvings on the surface, and three-hole grooves [18].

## 3. Traditional Chinese Medicine Theory

*Lonicerae japonicae flos* is sweet in taste and cold in nature, which is attributed to lung, heart, and stomach meridians. Its functions are clearing heat, resolving toxins, and eliminating external ailments. The clinical indication of *Lonicerae japonicae flos* is used for heat diseases, body heat, rashes, spots, sore heat toxins, and throat swelling pain, including carbuncles and pyocutaneous disease, pharyngitis, erysipelas, heat toxins, blood dysentery, and exogenous heat [17].

The TCM theory focuses on “The Yellow Emperor’s Classic of Internal Medicine” [19]. “Yin” and “Yang” and the “Five trespasses Elements” are the main TCM theories. It describes the physiological functions, pathological changes, and the relationship between an organ and the fundamental substances, consisting of “qi”, “blood”, and “body fluid” [20]. TCM balances the qi–blood–yin–yang in the body to maintain and keep humans healthy [21]. 

*Lonicerae japonicae flos* is a TCM, which possesses a variety of pharmacological functions, including anti-inflammatory, antibacterial, antifungal, and antioxidant properties. These are related to heat–clearing and detoxifying [22]. Based on the TCM theory, “wind”, “coldness”, “summer heat”, “dampness”, “dryness”, and “fire evils” are results of disrupted qi. It causes a deficiency of vital or internal energy and blood, leading to “Yin” and “Yang” imbalances. Thus, the pathogenic factors of eczema are “wind”, “dampness”, and “heat” [23]. 

*Lonicerae japonicae flos* is used to clear “damp heat” from the exterior. This aims to eliminate the accumulated damp and heat toxins within the body to restore normal bodily functions through pharmacological functions, such as anti-inflammatory and antioxidant properties (Table 2). They are associated with the extracted major flavonoids from *Lonicerae japonicae flos*, especially the chrysoeriol. This flavonoid is a secondary metabolite [24], which operates as a signal molecule, ultraviolet filter, and reactive oxygen species (ROS) scavenger [25].

Recently, Kim et al. reported the phenolic compounds in *Lonicera japonicae flos* and Chenpi distillation extract with antioxidant and anti-inflammation properties. These compounds exhibited a high binding affinity to DPPH and inhibited the anti-inflammation cytokines (COX-2 and iNOS), MAPK (JNK, ERK, and P38), and NF-kB pathways on skin disease [26]. 

**Table 2:** Pharmacological functions of *Lonicerae japonicae flos* for anti-inflammatory and antioxidant properties.

Chinese Medicinal Herb (CMH)	Metabolite (s)	Pharmacological Function (s)	Model/Dosage	Consequence	Reference
*Lonicerae japonicae flos*.	Flavonoids, phenolic compounds (polyphenolic).	Anti-inflammatory.	RAW264.7 cells; 2.5, 5 and 10 μg/mL (water extract).	Reduce the expression of proinflammatory mediators and inflammatory cytokines, such as cyclooxygenase inhibitors-2 and inducible nitric oxide synthase, through the suppression of the Janus kinase/signal transducers and activators of transcription-3-dependent Nuclear factor kappa-light-chain-enhancer of activated B cells pathway and the induction of Heme oxygenase-1 expression in Pseudorabies virus-infected RAW264.7 cells.	[27]
	Chlorogenic acid.	Anti-inflammatory.	Human neutrophils; 3, 10, and 30 μg/mL (ethanol extract).	Attenuates inflammatory reactions in the activated neutrophils, including superoxide anion generation, release of elastase, *CD11b* expression, chemotactic migration, cell adhesion, and neutrophil extracellular trap formation.	[28]
	Flavonoid (Loniceralanside A).	Anti-inflammatory.	Rat; 3.05 µM (ethanol extract).	Inhibits the release of β-glucuronidase induced by platelet-activating factor in rat polymorphonuclear leukocytes.	[29]
	Flavonoids, iridoids, triterpenoids, organic acids.	Anti-inflammatory, antioxidant.	C57BL/6 mice; 12.5, 25, and 50 mg/mL (water extract).	Relieve pressure-overload-induced heart failure following transverse aortic constriction, through increased heart antioxidant defense systems.	[30]
	Flavonoids, iridoids, triterpenoids, organic acids.	Anti-inflammatory.	BV-2 microglial cells; 0.5, 5, 2.5, 5, and 10 μg/mL (water extract).	Prevent lipopolysaccharide-induced activation of Nuclear factor kappa-light-chain-enhancer of activated B cells localization, and consequently reduce lipopolysaccharide-induced DNA–protein-binding activity of Nuclear factor kappa-light-chain-enhancer of activated B cells, leading to downregulation of proinflammatory mediators.	[31]
	Chlorogenic acid.	Anti-inflammatory.	Male Wistar rats; 231 μg/mL (water extract).	Suppresses the induction of nitric oxide production and *nitric oxide synthase* expression, which may have therapeutic potential for inflammatory diseases, including liver injury.	[32]
	Flavonoids, phenolic compounds (polyphenolic).	Anti-inflammatory, antioxidant.	HaCaT cells; 0.1, 0.25, 0.5, 0.75, 1, 1.25, 2, 2.5, 5, 7.5, and 10 µg/mL (methanol extract).	Polyphenolic compounds with antioxidant and anti-inflammatory effects since their molecular structural binding or affinity are suggested for various inflammation pathways.	[33]
	Flavonoids, iridoids, triterpenoids, organic acids.	Anti-inflammatory, antioxidant.	HaCaT cells; 0.1, 0.25 or 0.5 mg/mL (ethanol extract).	Exhibit protective effects on HaCaT cells against H_2_O_2_-induced oxidative stress through reactive oxygen species release, and inhibit skin damage against oxidative stress.	[34]

## 4. Extraction Techniques

The extraction techniques of chrysoeriol from *Lonicerae japonicae flos* mainly include solvent (water or ethanol) or maceration, reflux, and soxhlet, as well as ultrasonic-assisted extraction. They have some parameters summarized in Table 3, which include solvent type, time duration, advantages, and disadvantages.

### 4.1. Solvent (Liquid–Liquid) Extraction

Solvent (water or ethanol) penetrates into the solid matrix (*Lonicerae japonicae flos*), then solute dissolves in the solvents based on the “like dissolves like” principle. The solute is diffused out of the solid matrix, and the extracted solutes are collected finally [35]. Since chrysoeriol is a polar flavonoid, the solvent is usually ethanol or an ethanol–water mixture [36]. 

### 4.2. Maceration

Seventy percent methanol is also an efficient solvent for extracting chrysoeriol by maceration, but the *Lonicerae japonicae flos* must be used either fresh or dry [37]. This is carried out by soaking the *Lonicerae japonicae flos* with methanol or ethanol in a stoppered glass container. It is allowed to stand at room temperature for at least 3 days and shaken frequently [38].

### 4.3. Reflux and Soxhlet

Reflux is a high-temperature continuous extraction procedure using the soxhlet apparatus. *Lonicerae japonicae flos* are placed in a porous “thimble” paper, and the extraction solvent (e.g., methanol or ethanol) is heated in the bottom flask. The solvent vaporized into the sample thimble, and condensed in the condenser, then dripped back to the bottom flask [39]. It is a continuous reflux process to achieve the preconcentration of the extracted solutes.

### 4.4. Ultrasonic-Assisted Extraction (UAE)

This is a method using ultrasonic wave energy for the extraction. It accelerates the dissolution and diffusion of the cell ingredients, as well as propagation in the molecules of the medium. The high-purity product can be produced through continuous extraction [40]. 

**Table 3:** Common extraction techniques of chrysoeriol from *Lonicerae japonicae flos.*

	Active Ingredient	Solvent/Temperature/ Time Duration	Advantages/Disadvantages	References
Solvent (liquid–liquid) extraction.	Chrysoeriol.	Water or ethanol–water, 40 to 80 °C, 15 to 35 min.	Low equipment cost, wide extraction range, and simple operation. Time-consuming, compatibility issues, and potential contamination or cross-talk.	[41]
Maceration.	Chrysoeriol.	Methanol/ethanol, room temperature, several days or a few weeks at least.	Simple process, and no heat involved, suitability for thermal sensitive flavonoid. Low extraction yield, use of large volumes of solvents, long processing time, and further purification steps are required.	[42]
Reflux and Soxhlet.	Chrysoeriol.	Ethanol, boiling point of a solvent, 2 to 48 h.	High extraction efficiency. Long extraction time and consumption of large amounts of used solvents.	[43]
Ultrasonic-assisted extraction (UAE).	Chrysoeriol.	Ethanol, ultrasonic cleaning bath at 40 kHz, 40 to 60 °C, 10 to 60 min.	High efficiency and reduced extraction time. Energy and solvent consumption, present low extraction yields.	[44]

### 4.5. Example for Multistep Extraction of Chrysoeriol from Lonicerae japonicae flos 

Liu et al. reported that chrysoeriol was extracted from *Lonicerae japonicae flos* by the steps of alkali extraction and water precipitation, organic solvent extraction, membrane concentration, and macroporous resin adsorption. The extraction and separation time of this method is comparatively short, which has a high extraction rate (90%) and purity (98.6%); also, the preparation is simple and easy to operate. The steps are as follows [45]:

(i) Pulverize *Lonicerae japonicae flos*, and use the ultrasonic extraction for 0.5–3 h, then filter and concentrate to neutral with acid for adjusting pH. Suspension liquid is produced through precipitation by adding distilled water;
(ii) Add ethyl acetate for extraction, and combine the extraction liquid after the suspension liquid is added to chloroform extraction;
(iii) The extracted liquid then undergoes microfiltration, ultrafiltration, and nanofiltration successively in the multifunctional membrane separating device;
(iv) Wash the extractant with deionized water until colorless, discard the water portion, and use the 30% aqueous ethanolic solution gradient elution again, from 10% and incremented to 90%, then collect elutriant;
(v) Evaporate the ethanol, concentrate, and dry with 50% methanol. The extractant is cooled and stands overnight for crystallization to afford the chrysoeriol crude product;
(vi) Repeat the extraction steps by using ethyl acetate, methanol, acetone, and chloroform or recrystallization again to obtain the pure product of chrysoeriol.

## 5. Chrysoeriol

### 5.1. Source

Chrysoeriol is a CMH and dietary flavonoid that exists in *Lonicerae japonicae flos* and other different foods, such as wild celeries (*Apium graveolens* L.), tartary buckwheats (*Fagopyrum tataricum Gaertn.*), hard wheat (*Triticum durum*), oats (*Avena sativa*), and common thymes (*Thymus vulgaris*). Chrysoeriol may also be considered a flavonoid lipid molecule for the prevention of fat oxidation and the protection of vitamins and enzymes [46].

### 5.2. Structure

**Figure 2:**
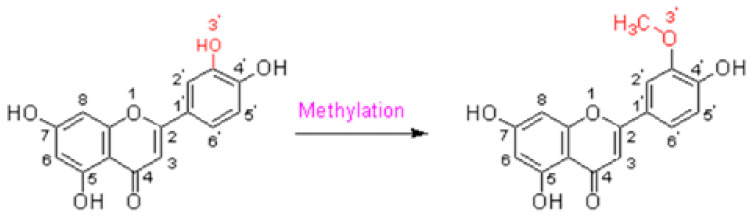
Methylation of luteolin to chrysoeriol.

Chrysoseriol (4′,5,7-trihydroxy-3′-methoxyflavone, C_16_H_12_O_6_) is a major flavone and secondary metabolite from *Lonicerae japonicae flos*. It is light yellow to yellow in color. This is the 3′-O-methyl derivative of luteolin, belonging to the 3′-O-methylated flavonoids. It consists of a trihydroxyflavone and a monomethoxyflavone. The function is similar to a luteolin. They are flavonoids with methoxy groups attached to the C3′ atom of the luteolin (Figure 2) [47,48]. 

The O-methylation is driven by a catalyst, O-methyltransferases (OMTs). This is an important modification of flavonoids for improving the transport efficiency across membranes and metabolic stability in mammalian cells. Wu X et al. identified that the generation of chrysoeriol from luteolin can be catalyzed by a rice-derived 3′-OMT, which has a high regio-specificity and activity toward flavonoids in vitro, such as *Escherichia coli* [49]. 

### 5.3. Structure–Activity Relationship

#### 5.3.1. Antioxidant

The antioxidant activity of chrysoeriol depends upon the arrangement of functional groups about the configuration, substitution, and total number of hydroxyl groups. It involves (i) radical scavenging and (ii) metal ion chelation ability [50,51].

**Figure 3:**
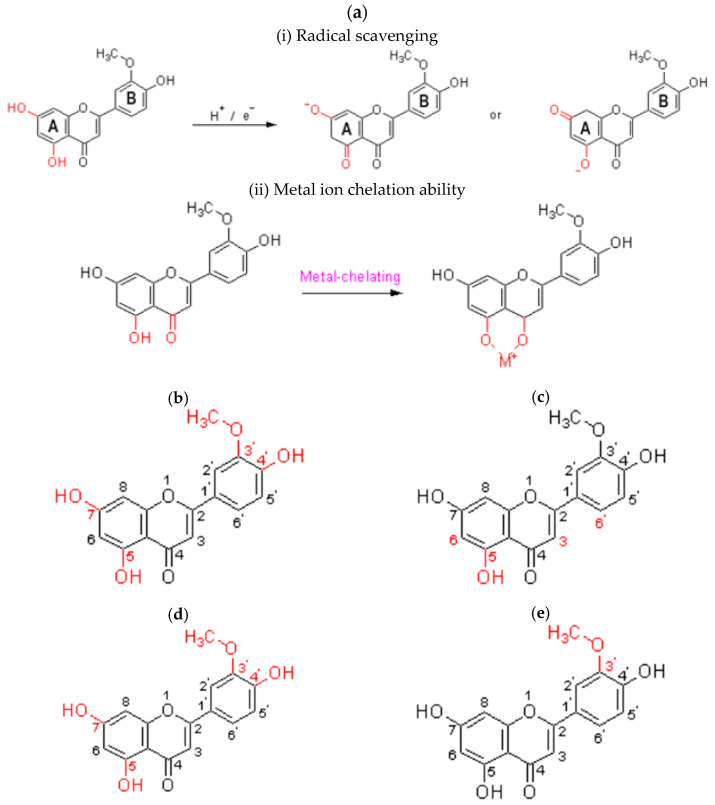

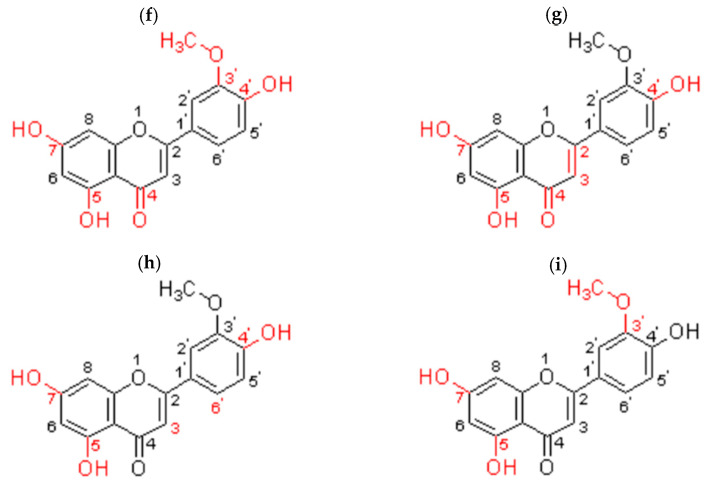
Structure–activity relationship of (**a**) antioxidant, (**b**) anti-inflammatory, (**c**) anticancer, (**d**) antidiabetic, (**e**) antiarthritis, (**f**) antimicrobial, (**g**) antithrombotic, (**h**) antihyperlipidemic, and (**i**) antinociceptive on chrysoeriol.

(i) The “A” ring is the most suitable for scavenging ROS because it releases hydrogen or donates an electron from the hydroxyl group to form a stable flavonoid radical (Figure 3a(i)) [52], but the “B” ring has the 3’-OMT, which is a steric hindrance. (ii) Trace metal (M^+^) such as metal-chelating (Figure 3a(ii)) and metal-stabilizing properties occur in the “A” ring, since there may be a “hydrogen bond” formation between the hydroxyl and ketone groups. This is similar to the quercetin in terms of iron-chelating and iron-stabilizing [53].

#### 5.3.2. Anti-Inflammatory

Hydroxyl groups are indispensable for the anti-inflammatory function of chrysoeriol. The hydroxyl group at the C5 and C4′ positions enhances its function while the hydroxyl group at C7 and methoxyl group at the C3′ positions attenuate their activity (Figure 3b) [54].

#### 5.3.3. Anticancer

The anticancer activity of chrysoeriol is based on the C6-C3-C6 skeleton, and it contains the hydroxyl group at C5 in its structure (Figure 3c), which has a lower cytotoxic activity [55].

#### 5.3.4. Antidiabetic

Cutaneous complications occur from antidiabetic therapy, which might be caused by insulin therapy, lipoatrophy, erythema, local infections, subcutaneous nodules, and allergies [56].

The absence of the C-2-C-3 double bond and ketonic group at C-4 reduced the xanthine inhibitory activities of oxidase, α-glucosidase, and Dipeptidyl peptidase IV (DPP-4) inhibitory activities. Meanwhile, the hydroxyl group at the position of C-4′ also enhances the DPP-4 inhibitory activities (Figure 3d). The DPP-4 inhibitors are one of the newest therapeutic agents against type 2 diabetes mellitus [57,58]. 

This is a serine exopeptidase hormone, which degrades two major gut incretin hormones to stimulate insulin release. The glucagon-like peptide-1 (GLP-1) and glucose-dependent insulinotropic polypeptide (GIP) lead to a very short half-life (approximately 2 min) of the hormones for regulating plasma insulin levels in the human body [59,60]. 

#### 5.3.5. Antiarthritis

The 4′-OH hydrogroup enhances α-glucosidase inhibition activity (Figure 3e) [61] to acarbose. It decreases the rheumatoid arthritis risk in diabetic patients. Arthritis and the expression of proinflammatory cytokines, including TNF-α, IL-6, and IL-17, were decreased in the paw tissues [62]. 

#### 5.3.6. Antimicrobial

Chrysoeriol has a great inhibition effect on the Gram-positive bacteria except *S. aureus* CCARM 0027 (MSSA), *Enterococcus faecalis* 19433, and *Enterococcus faecalis* 19434, as well as against *Proteus hauseria* NBRC 3851 [63]. The antimicrobial function depends on the hydroxyl group in C4′, C5, and C7 since the hydroxylation boosts the activity. A hydroxyl group at C5 can form an intramolecular hydrogen bond with the carbonyl group at position C4, resulting in more electron delocalization inside the molecule. However, the methoxylation at C3′ may decrease an antibacterial action, because it cannot establish the intramolecular hydrogen bond (Figure 3f) [64].

#### 5.3.7. Antithrombotic

Antithrombotic alterations have been reported in atopy and fibrin clot function in atopic patients. The plasma fibrin clot properties associated with reduced efficiency of fibrinolysis can be detected in atopic dermatitis patients, which might represent a novel mechanism that modulates a hemostatic balance in atopy [65].

Hydroxyl groups on C5 and C7 are beneficial for the antithrombin effect. C=O on C4 and C=C on C2-C3 are essential for thrombin inhibition, but the single hydroxyl group at the C5 site should weaken the antithrombin properties (Figure 3g) [66]. 

#### 5.3.8. Antihyperlipidemic

Chrysoeriol can regulate the imbalance of lipid metabolism by inhibiting lipid peroxidation and endogenous lipid biosynthesis and promoting lipid redistribution and exogenous lipid metabolism, significantly reducing triglyceride, total cholesterol, and low-density lipoprotein levels [67]. The antihyperlipidemic properties of chrysoeriol are related to the number of hydroxyl groups at C5, C7, and C4′ in the C6-C3-C6′ skeleton (Figure 3h). These significantly regulate the lipid metabolism through free radical scavenging activity [68].

#### 5.3.9. Antinociceptive

Antinociceptive activity has a relationship with the opioid receptors in the nervous system. It is used as a complementary treatment for inflammatory disorders of the skin [69].

The presence of dihydroxy groups at C5 and C7 positions can significantly contribute to the antinociceptive activities of chrysoeriol because of the formation of free radical scavenging. It decreases the topical activity since the methoxy group is at the C3 position (Figure 3i) [70].

### 5.4. Pharmacological Functions

Chrysoeriol is established to have a variety of pharmacological functions, including antioxidant [48], anti-inflammatory [71], anticancer [72], antidiabetic [73], antiarthritis [74], antimicrobial [75], antithrombotic [76], antihyperlipidemic [77], and antinociceptive [78] (Table 4), as well as interferes in certain disease-related progression pathways [71]. 

### 5.5. Pharmacokinetic and Pharmacodynamic Effects

Pharmacokinetics plays a vital role in understanding the drug’s effectiveness, time frame of reactions, and eventual expulsion from the body. It consists of four important stages: absorption, distribution, metabolism, and excretion (ADME) [79]. Chrysoeriol is structurally diverse and among the most ubiquitous TCM groups. The pharmacokinetics of chrysoeriol are associated with pharmacological and toxicological profiles. These profiles are useful to evaluate the potential risks and benefits for human health [80] because chrysoeriol has a variety of pharmacological functions for some diseases (as discussed above in Section 5.4). Chen et al. studied the pharmacokinetic effect of chrysoeriol after oral administration of *Flos Chrysanthemi* extract (FCE) in rats. The HPLC system was successfully validated and applied to the oral administration of FCE to rats with or without cathodol-O-methyltransferase inhibitor, entacapone. The concentration of chrysoeriol in the plasma was significantly reduced [81]. Li et al. identified that chrysoeriol has good stability and pharmacokinetic behavior properties in molecular dynamic simulation of xanthine oxidase (XO) through the formation of hydrogen bonding and hydrophobic interactions, as well as absorption, distribution, metabolism, and excretion (ADME) prediction. This is expected to induce hyperuricemia and increase the level of superoxide free radicals in blood for the treatment of gout [82].

Pharmacodynamics is the study of drugs for interacting with biological structures or targets at the molecular level to induce a change in how the target molecule functions concerning subsequent intermolecular interactions [83]. These interactions result in competition for receptor binding sites or alter photoreceptor response [84], for example, the structure–activity relationship of chrysoeriol at a particular concentration to occupy the receptor (as discussed above in Section 5.3). 

## 6. Cutaneous Delivery System

Flavonoids have been demonstrated as a suitable agent in the treatment of skin disorders. Domaszewska-Szostek et al. discovered flavonoids that can slow down or prevent aging-associated deterioration of skin appearance and its function. This is related to the target cellular pathways for regulating cellular senescence and senescence-associated secretory phenotype [85]. However, most flavonoids are lipophilic in nature, and poor water solubility invariably leads to limited oral bioavailability [86].

Chrysoeriol is a very hydrophobic molecule, practically insoluble in water, and relatively neutral. It is lipophilic; thus, the cutaneous route is the best delivery approach, which depends on the solubility and permeability of chrysoeriol across the skin barrier [87]. Lai et al. indicated that chrysoeriol efficiently bound in the active site cavity, and was able to inhibit the activity of c-Met and Vascular endothelial growth factor receptor 2 (VEGFR2) and may serve as the leading compound for novel drug development, especially in the tumorigenesis of various types of cancer [88]. In fact, VEGFR2 is a primary responder to vascular endothelial growth factor signal and thereby regulates endothelial migration and proliferation; hence, chrysoeriol can also be expressed in endothelial cells of developing capillaries, thoracic duct, great vessels, hepatic sinusoids, epidermis, and mesothelial for the treatment of skin disease [89].

Wu et al. demonstrated that chrysoeriol ameliorates TPA-induced ear edema in mice through JAK2/STAT3 and IκB/p65 NF-κB pathways. As chrysoeriol decreased the production of NO and prostaglandin E2, which inhibited the phosphorylation of inhibitor of κB (Ser32), p65 (Ser536), and Janus kinase 2 (Tyr1007/1008). It also decreased nuclear localization of p50, p65, and STAT3, and downregulated mRNA levels of proinflammatory cytokines IL-6, IL-1β, and TNF-α, which are transcriptionally regulated by NF-κB and STAT3 in the RAW264.7 cells model [71].

**Figure 4:**
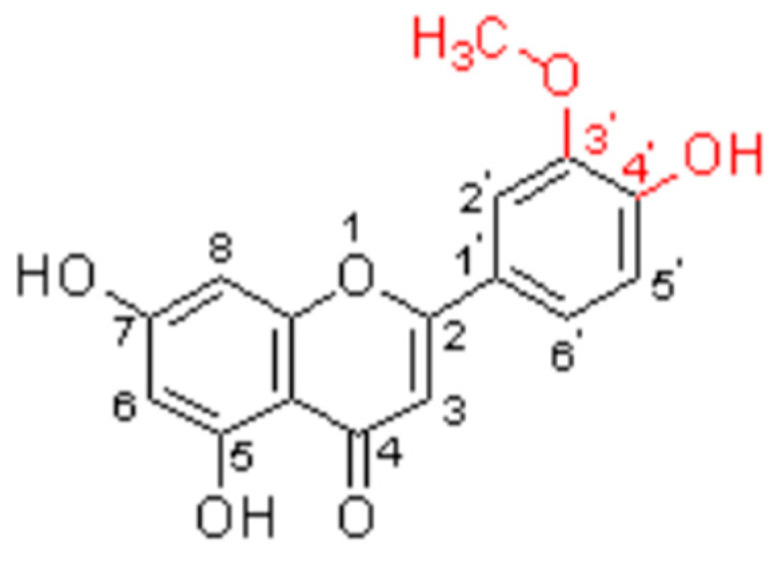
Structure–activity relationship of melanogenesis on chrysoeriol.

Recently, Oh et al. identified the significance of the C4′-OH group and C3′ methoxylation for melanogenesis in the structure of chrysoeriol (Figure 4). It promotes melanogenesis in B16F10 cells by upregulating the expression of melanogenic enzymes through the MAPK, phosphatidylinositol 3-kinase (PI3K)/AKT, PKA, and Wnt/β-catenin signaling pathways. This can prevent hypopigmentation disorders [90].

## 7. Conclusions

Chrysoeriol is a flavonoid extracted from *Lonicerae japonicae flos*. It is a second metabolite from this CMH with a wide range of pharmaceutical functions that have a structure–activity relationship. The cutaneous route is the best delivery approach for chrysoeriol across the skin barrier, but it depends on its structure, solubility, and permeability of chrysoeriol. However, much more work needs to be carried out, such as the toxicity, dosage, and safety assessments of chrysoeriol on the human epidermis to fight against skin diseases.

## Figures and Tables

**Table 1:** List of flavonoids, iridoids, triterpenoids, and organic acids from *Lonicerae japonicae flos* [3].

Flavonoids	(1) quercetin, (2) rutin, (3) luteolin-7-*O*-β-d-glucopyranoside, (4) kaempferol-3-*O*-β-d-glucopyranoside, (5) apigenin-7-*O*-α-l-rhamnopyranoside, (6) chrysoeriol-7-*O*-β-d-glucopyranosyl, (7) luteolin-3′-l-rhamnoside, (8) luteolin, (9) flavoyadorinin-B, (10) rhoifolin, (11) quercetin-3-*O*-β-d-glucopyranoside, (12) 3′-methoxy luteolin, (13) 5,3′-dimethoxy luteolin, (14) luteolin-5-*O*-β-d-glucopyranoside, (15) apigenin, (16) isorhamnetin-3-*O*-β-d-glucopyranoside, (17) hyperoside, (18) quercetin-7-*O*-β-d-glucopyranoside, (19) kaempferol-3-*O*-β-d-rutinoside, (20) isorhamnetin-3-*O*-β-d-rutinoside, (21) 5-hydroxyl-3′,4′,7-trimethoxy flavone, (22) 5-hydroxyl-6,7,8,4′-tetramethoxy flavone, (23) corymbosin, (24) 5-hydroxyl-7,4′-dimethoxy flavone, (25) lonicerin, (26) 5,7,3′,4′,5′-pentamethoxy flavone, and (27) 5,4′-dihydroxy-3′,5′-dimethoxy-7-β-d-glucoxy-flavone
Iridoids	Consist of iridoid glucosides, secoiridoid glycosides, and N-contained iridoid glycosides.iridoid glucosides: (1) loganin, (2) 8-epiloganin, (3) loganic acid, (4) 8-epiloganic acid, and (5) ketologanin. secoiridoid glycosides: (6) secologanin, (7) secologanoside, (8) secoxyloganin, (9) secologanin dimethyl acetal, (10) secologanoside-7-methyl ester, (11) secologanic acid, (12) sweroside, (13) 7-*O*-ethylsweroside, vogeloside, (14) 7-epi-vogeloside, secoxyloganin-7-butyl ester, (15) kingiside, (16) 8-epikingiside, (17) 7*α*-morroniside, (18) 7*β*-morroniside, (19) dehydromorroniside, (20) 7-hydroxy-methyl-vogeloside, (21) (*Z*)-aldosecologanin, (22) (*E*)-aldosecologanin, (23) loniaceticiridoside, (24) lonimalondialiridoside, (25) 6′-*O*-acetylvogeloside, (26) 6′-*O*-acetylsecoxyloganin, (27) loniceracetalide A, (28) loniceracetalide B, (29) adinoside A, (30) stryspinoside, (31) secologanoside A, (32) dimethyl secologanoside, (33–36) loniphenyruviridoside A~D, (37) centauroside, (38) loniceranan A, (39) loniceranan B, (40) loniceranan C, (41) ethyl secologanoside, (42) demethylsecologanol, (43) harpagide, (44) harpagoside, (45) 6′′-*O*-*β*-glucopyranosylharpagoside, (46) (7*β*)-7-*O*-methyl morroniside, (47) lonicerjaponin A, and (48) lonicerjaponin B. N-contained iridoid glycosides: (49) serinosecologanin, (50) threoninosecologanin, (51) lonijaponinicotinosides A, (52) lonijaponinicotinosides B, (53) lonijapospiroside A, (54) L-phenylalaninosecologanin B, (55) L-phenylalaninosecologanin C, (56) dehydroprolinoylloganin A, (57–59) lonijaposides A-C, (60–70) lonijaposides D-N, and (71–83) lonijaposides O-W.
Triterpenoids	(1) limonin, (2) ursolic acid, (3) oleanolic acid triterpenoid saponins, (4) hederagenin triterpenoid saponins, (5) oleanolic acid, 3-*O*-β-d-glucopyranosyl-(12)-α-l-arabinopyranosyl oleanolic acid-28-*O*-β-d-glucopyranosyl-(16)-β-d-glucopyranoside, (6) oleanolic acid 28-*O*-α-l-rhamnopyranosyl-(12)-[*β*-D-xylopyranosyl(16)]-β-d-glucopyranosyl ester, (7) loniceroside E, hederagenin 3-*O*-α-l-arabinopyranoside, (8) loniceroside D, (9) loniceroside A, (10) loniceroside B, (11) loniceroside C, (12) 3-*O*-β-d-glucopyranosyl(14)-β-d-glucopyranosyl(13)-α-l-rhamnopyranosyl(12)-α-l-arabinopyranosyl-hederagenin-28-*O*-β-d-glucopyranosyl(16)-β-d-glucopyranosyl ester, (13) hederagenin-3-*O*-α-l-rhamnopyranosyl(12)-α-l-arabinopyranoside, (14) 3-*O*-α-l-rhamnopyranosyl(12)-α-l-arabinopyranosyl-hederagenin-28-*O*-β-d-xylopyranosyl(16)-β-d-glucopyranosyl ester, (15) 3-*O*-α-l-rhamnopyranosyl(12)-α-l-arabinopyranosyl-hederagenin-28-*O*-β-d-glucopyranosyl(16)-β-d-glucopyranosyl ester, (16) 3-*O*-α-l-rhamnopyranosyl(12)-α-l-arabinopyranosyl-hederagenin-28-*O*-β-d-rhamnopyranosyl(12)-[*β*-D-xylopyranosyl(16)]-β-d-glucopyranosyl ester, and (17) 3-*O*-β-d-glucopyranosyl(13)-α-l-rhamnopyranosyl(12)-α-l-arabinopyranosyl-hederagenin-28-*O*-β-d-glucopyranosyl(16)-β-d-glucopyranosyl ester.
Organic acids	(1) myristic acid, (2) palmitic acid, (3) 2(*E*)-3-ethoxy acrylic acid, (4) ethyl laurate, (5) protocatechuic acid, (6) abscisic acid, (7) 3-(3, 4-dihydroxyphenyl) propionic acid, (8) caffeic acid, (9) ferulic acid, (10) caffeic acid methyl ester, (11) methyl 4-*O*-β-d-glucopyranosyl caffeate, (12) caffeic acid ethyl ester, (13) cinnamic acid, (14) 4-hydroxycinnamic acid, (15) methyl 4-hydroxycinnamate, (16) 1-*O*-caffeoylquinic acid, (17) 3-*O*-caffeoylquinic acid, (18) 4-*O*-caffeoylquinic acid, (19) 5-*O*-caffeoylquinic acid, (20) 3-*O*-caffeoylquinic acid methyl ester, (21) 3-*O*-caffeoylquinic acid ethyl ester, (22) 3-*O*-caffeoylquinic acid butyl ester, (23) 4-*O*-caffeoylquinic acid methyl ester, (24) 5-*O*-caffeoylquinic acid butyl ester, (25) 5-*O*-caffeoylquinic acid methyl ester, (26) 3,5-*O*-dicaffeoylquinic acid, (27) 3,4-*O*-dicaffeoylquinic acid, (28) 4,5-*O*-dicaffeoylquinic acid, (29) 3,5-*O*-dicaffeoylquinic acid methyl ester, (30) 3,5-*O*-dicaffeoylquinic acid butyl ester, (31) 3,5-*O*-dicaffeoylquinic acid ethyl ester, (32) 3,4-*O*-dicaffeoylquinic acid methyl ester, (33) 3,4-*O*-dicaffeoylquinic acid ethyl ester, (34) 4,5-*O*-dicaffeoylquinic acid methyl ester, (35) 3,4,5-*O*-tricaffeoylquinic acid, (36) vanillic acid, (37) 4-*O*-β-d-(6-*O*-benzoylglucopyranoside), (38) (−)-4-*O*-(4-*O*-β-d-glucopyranosylcaffeoyl) quinic acid, (39) (−)-3-*O*-(4-*O*-β-d-glucopyranosylcaffeoyl) quinic acid, (40) (−)-5-O-(4-*O*-β-d-glucopyranosylcaffeoyl) quinic acid, and (41) dichlorogelignate.

**Table 4:** The pharmacological effect of chrysoeriol on different diseases.

Pharmacological Function (s)	Model/Dosage	Consequence	Reference
Antioxidant.	Human aortic smooth muscle cells; 5 and 10 μM.	The downstream signal transduction pathways of platelet-derived growth factor receptor beta, including extracellular signal-regulated protein kinases 1 and 2, p38, and Protein kinase B phosphorylation for preventing and treating vascular diseases.	[48]
Anti-inflammatory.	RAW264.7 cell, and TPA (12-*O*-tetradecanoylphorbol-13-acetate)-induced ear edema mouse; 0, 10, 20 μM.	Chrysoeriol ameliorated TPA-induced ear edema in mice and inhibition of JAK2/STAT3 and IκB/p65 NF-κB pathways.	[71]
Anticancer.	A549 cells and xenografted mice; 7.5, 15, and 30 μM.	The expression of *LC3-phosphatidylethanolamine conjugate* and Beclin-1 are significantly upregulated, and also induce sub-G1/G0 cell cycle arrest, as well as inhibit the migration and invasion of the A549 cells.	[72]
Antidiabetic.	Diabetic rats; 20 mg/kg.	The level of glucose reduced with the decreased in the enzyme HbA1 in diabetic rats.	[73]
Antiarthritis.	Rheumatoid arthritis-fibroblast-like synoviocytes; 5, 10, 20, 40, and 80 μM.	Suppress hyperproliferation of, and evoke apoptosis in, Interleukin-6/receptor-stimulated rheumatoid arthritis-fibroblast-like synoviocytes by its ability to cleave caspase-3 and caspase-9.	[74]
Antimicrobial.	*Fusarium graminearum* and *Pythium graminicola*; 0.1, 0.5, 1 μM.	High inhibition rate and limiting the growth of pathogens of *Fusarium graminearum* and *Pythium graminicola*.	[75]
Antithrombotic.	SW872 Human liposarcoma cell; 25, 50, 100, and 200 μM.	Inhibition of pancreatic lipase, cholesterol esterase, adipocytes lipid uptake, and antithrombotic activity, which act as a potential source for future antiatherosclerotic drug discovery.	[76]
Antioxidant, antihyperlipidemic.	Wistar rats; 800 mg/kg.	Reduce triglyceride, low-density lipoprotein, cholesterol, and total cholesterol, as well as increase the high-density lipoprotein cholesterol level for improving lipid metabolism.	[77]
Antinociceptive.	Male BALB/c mice; 200 mg/kg or 400 mg/kg.	Based on the molecular docking simulations, chrysoeriol interacts with the α2-adrenergic receptor to exert its analgesic.	[78]

## Abbreviations

ADME	Absorption, distribution, metabolism, and excretion
CMH	Chinese medicinal herb
ChP	Chinese Pharmacopoeia
DPP-4	Dipeptidyl peptidase IV
ERK1/2	Extracellular signal-regulated protein kinases 1 and 2
FCE	*Flos chrysanthemi* extract
GLP-1	Glucagon-like peptide-1
GIP	Glucose-dependent insulinotropic polypeptide
HO-1	Heme oxygenase-1
*IL-6*	*Interleukin-6*
*IL-7*	*Interleukin-7*
IκB	IκB kinase
JAK2	Janus kinase 2
JAK/STAT/3	Janus kinase/signal transducers and activators of transcription-3
LC3II	LC3-phosphatidylethanolamine conjugate
LPS	Lipopolysaccharide
NF-κB	Nuclear factor kappa-light-chain-enhancer of activated B cells
65 kDa	p65
PI3K	Phosphatidylinositol 3-kinase
PAF	Platelet-activating factor
PDGFRß	Platelet-derived growth factor receptor beta
PMN	Polymorphonuclear leukocyte
PKA	*Protein kinase A*
AKT	*Protein kinase B*
PRV	Pseudorabies virus
ROS	Reactive oxygen species
STAT3	Signal transducers and activators of transcription 3
TPA	Tissue plasminogen activator
TCM	Traditional Chinese medicine
